# A case of hemophagocytic lymphohistiocytosis after BNT162b2 COVID-19 (Comirnaty®) vaccination

**DOI:** 10.1097/MD.0000000000031304

**Published:** 2022-10-28

**Authors:** Yoshitaka Shimada, Yasushi Nagaba, Hiroyuki Okawa, Kaori Ehara, Shinya Okada, Hiroaki Yokomori

**Affiliations:** a Department of Internal Medicine, Kitasato University Medical Center, Saitama, Japan; b Division of Pathology, Kitasato University Medical Center, Saitama, Japan.

**Keywords:** CKD, COVID-19, hemophagocytic lymphohistiocytosis, vaccination, elderly patient

## Abstract

Coronavirus disease (COVID-19), an infectious disease caused by the severe acute respiratory syndrome coronavirus 2 virus, was reported in Wuhan of China in December 2019. The world is still in a state of pandemic owing to COVID-19. COVID-19 vaccines help our bodies develop immunity against the virus that causes COVID-19 without having to get the illness. Herein, we describe a rare case of a critical disorder, hemophagocytic lymphohistiocytosis (HLH), in a patient with nephritic sclerosis associated with hypertension, following mRNA COVID-19 vaccination. HLH is a life-threatening hyperinflammatory syndrome caused by aberrantly activated macrophages and cytotoxic T cells that may rapidly progress to terminal multiple organ failure.

**Patient concerns:**

An 85-year-old Japanese woman with chronic renal failure and hypertension was included in this study. Routine laboratory investigations provided the following results: white blood cell (WBC) count, 4.6 × 10^9^/L; hemoglobin (Hb), 8.1 g/dL; platelet count, 27 × 10^9^/L; blood urea nitrogen 48.9 mg/dL, and serum creatinine 3.95 mg/dL. The patient developed malaise, vomiting, and persistent high fever (up to 39.7°C) on the 12^th^ day after receiving the second dose of the vaccine. Initial evaluation revealed neutropenia. The total WBC count was 0.40 × 10^9^/L (Neutrophils 0, Lymphocytes 240/μ, blast 0%); Hb 9.0 g/dL, platelet count 27 × 10^9^/L; and, *C* Reactive Protein 9.64 mg/dL.

**Diagnosis:**

Further tests showed hyperferritinemia (serum ferritin 2284.4 μg/L). Bone marrow examination revealed haemophagocytosis. A provisional diagnosis of HLH associated with the Comirnaty^®^ vaccination was made based on the HLH-2004 diagnostic criteria.

**Interventions:**

The patient was treated with granulocyte colony-stimulating factor and 500 mg methylprednisolone.

**Outcomes:**

A significant improvement was observed in the patient’s condition; the abnormal laboratory results resolved gradually, and the patient was discharged.

**Lessons:**

This case serves to create awareness among clinicians that HLH is a rare complication of COVID-19 vaccination and should be considered, especially in patients with a history of chronic renal failure and hypertension.

## 1. Introduction

The coronavirus disease 2019 (COVID-19) pandemic has caused a sudden and significant increase in hospitalizations of patients who develop pneumonia with multiorgan disease. It is caused by the novel severe acute respiratory syndrome coronavirus 2 (SARS-CoV-2). SARS CoV-2 infection may be asymptomatic or it may cause a wide spectrum of symptoms, ranging from mild symptoms of upper respiratory tract infection to life-threatening complications, which could culminate in death.^[1]^ In this current global pandemic of COVID-19, safe and effective vaccination is the most cost-effective solution to reduce the global burden of COVID-19.^[2]^ After the discovery of the SARS-CoV-2 genetic sequence in January 2020, multiple mRNA vaccines for COVID-19 were developed to target the spike protein.^[2]^ The development of the mRNA-based vaccine to prevent COVID-19 infection was a success, with no significant health consequences. Only minor side effects such as redness, pain, and swelling have been reported with these vaccine candidates. In addition, systemic symptoms of fatigue, fever, headache, myalgia, and arthralgia have also been observed during the window period of the first 24 to 48 hours of vaccination.^[3]^

Hemophagocytic lymphohistiocytosis (HLH) is a rare but often fatal dysregulated hyperimmune response that clinically resembles sepsis. It has been classified as either familial with known genetic defects in lymphocyte cytotoxicity or as acquired/secondary HLH (sHLH). sHLH in adults is usually secondary to an infection, malignancy, or autoimmune disease, although HLH triggered by conventional vaccination such as influenza has been reported.^[4]^ HLH can also rapidly progress to multiple organ failure and, if untreated, is often fatal.

Some cases of HLH following the ChAdOx1 AstraZeneca vaccine,^[5,6]^ inactive vaccine^[7]^ and BNT162b2 COVID 19 vaccine (Comirnaty^®^, BioNTech, and Pfizer) (9–13) have also been reported.

Herein, we describe a rare case of another critical disorder, HLH, in a patient with chronic renal failure associated with hypertension after Comirnaty^®^ vaccination.

## 2. Case presentation

**Figure 1.**
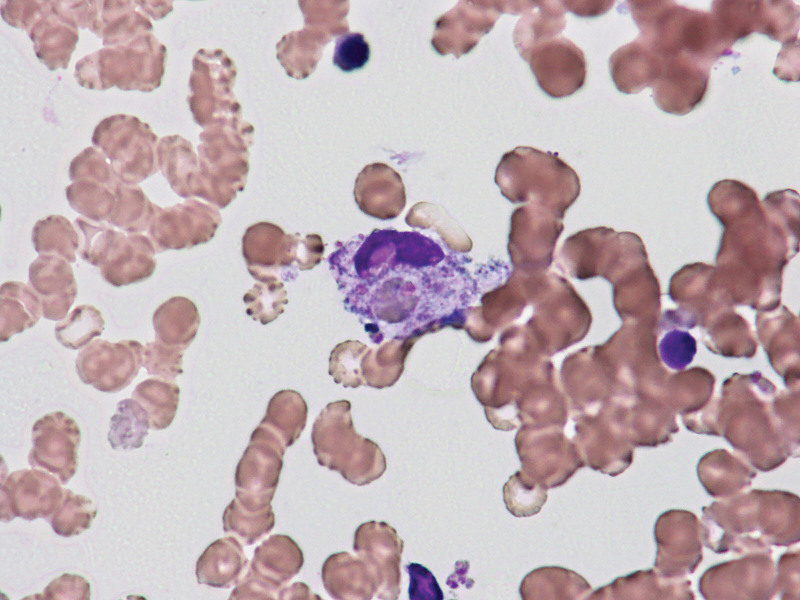
Bone marrow aspiration. Hemophagocytosis in the bone marrow: the arrows indicate phagocytosis erythrocytes (×200, Hematoxylin and Eosin).

An 85-years-old Japanese woman with a 10-years history of nephrosclerosis with hypertension presented herself to our hospital with a history of fever, temperature > 38°C for 7 days, and nonspecific fatigue. The patient was admitted 12 days after the first COVID-19 vaccination with Comirnaty^®^. Preliminary examination of the patient revealed the following findings: body temperature, 39°C; blood pressure, 120/80 mm Hg; heart rate 90 beats/minute, SpO_2_ 90% to 95%. No palpable or superficial lymph nodes were observed. No abnormalities were found on auscultation. Laboratory data showed white blood cell 400 × 10^3^/μL (Neut 0% Lymph 60%, Mono 34%, Blast 2%), Hb 8.1 g/dL, Plt 28.7 × 10^4^/μL, *C* Reactive Protein 9.64 mg/dL. Other findings were elevated levels of serum lactate dehydrogenase, 904 U/L (124–222), soluble IL-2 R 1450U/mL (1–613), IL-6 37 pg/mL (<7), and ferritin 2284 ng/ml (10–80); the reference intervals are given in parentheses (Table 1). Chest and abdominal computed tomography (CT) revealed no bilateral lung consolidation, pleural effusion, or splenopmegaly. At this stage, bone marrow aspiration was performed, and microscopic examination revealed agranulocytosis and anemia. The bone marrow aspirate shows two histiocytes (arrows) phagocytosing erythrocytes (Fig. 1). Flow cytometry was performed on bone marrow specimens using 3 - or 4-color antibody panels against a variety of lymphoid LLA/CD45 and myeloid MMA/CD38; however, these were not detected.

**Table 1.** Laboratory data.

Blood count	Blood chemistry	Serological examination	Urinalysis
WBC 400 × 10^3^/μL (4000–9000)	T.Bil 1.1 mg/dL (0.2–1.0)	IgG 934 mg/dL (870–1700)	Gravity 1.010 (1.005–1.0)
Neut 0 % (40–75) Stab 0 % (1.0–7.0)	AST 24 U/l (10–35)	IgA 416 mg/dL (110–410)	pH 5.0 (5.0–7.5)
Seg 0 % (34–70) Lymph 60 % (18–49)	ALT 7 U/l (5–40)	IgM 50 mg/dL (46–260)	Sugar (–) (–)
Mono 34.0 % (2.0–10)Eosino 2.0 % (0–8)	LDH 265 U/l (124–222)	CH50 29.2 U/mL (30.0–46.0)	Protein (++) (–)
Baso 2.0 % (0) Blast 2.0% (0)	ALP 68 U/l (38–113)	C3 60 mg/dL (65–135)	Occult blood (–)
RBC 259 × 10^3^/μL (400–900)		C4 34 mg/dL (13–35)	Keton(–)(–)
Hb 8.1 g/dL (11.5–15.0)	CK114 U/l (44–170)	ANA ×40 (<×40)	Bil(–)(–)(–)
Hct 24.8 (33.0–45.0)	T-Cho 174 mg/dL (120–219)	P-ANCA < 0.5 IU/mL (<3.5)	Uro(–)(–)
MCV 96 fl (85–95)	TG 83 mg/dL (30–149)	C-ANCA < 0.5 IU/mL (<3.5)	WBC(–)(–)
MCH 31.3 pg (28.0–33.0)	TP 7.6 g/dL (6.5–8.1)	Anti-GMM < 1.3 (<7.0)	Urinary segment Tubular epithelium 1–4/HPF
MCHC 32.6 % (30.0–36.0)	Alb 3.9 g/dL (3.8–5.2)	Soluble IL-2 R 1450 U/mL (121–613)	
Plt 28.7 × 10^3^/μL (140–340)	BUN 62.6 mg/dL (8.0–22.0)	IL-6 37 pg/mL (<7)	
Reti 15 % (4–19)	Cr 3.53 mg/dL (0.40–0.80)	Ferritin 2284.4 ng/mL (4.0–87.0)	
Coagulation system	UA 4.1 mg/dL (2.4–7.0)	Erythropoietin 25.1 mU/mL (4.2–23.7)	
PT-INR 1.07 (0.85–1.15)	eGFR 10.1 mL/min	HSV IgG + 56.8 (<2.0)	
APTT 43.9 Sec (25–36)	Na 130 mEq/L (135–146)	HSV-IgM < 0.8 (<0.80)	
Fib 551 mg/dL (155–415)	K 5.1 mEq/L (3.4–4.8)	CMVIgG 228 AU/mL (<6.0)	
FDP 5.4 μg/mL (<5)	Cl 106 mEq/L (98–108)	CMVIgM0.10 S/CO (<0.85)	
DD 1.4 μg/mL (<1)	Ca 9.5 mg/dL (8.4–10.4)	EBVIgG ×160 (<×10)	
	CRP 9.64 mg/dL (<0.3)	EBVIgM < ×10 (<×10)	
		EBNA ×20 (<×10)	

ALB = albumin, ALP = alkaline phosphatase, ALT = alanine aminotransferase, ANA = anti-nuclear antibody, Anti-GMM = anti-glomerular basement membrane antibody, APTT = partial thromboplastin time, AST = aspartate aminotransferase, BUN = blood urea nitrogen, C3 = complement component C3, C4 = complement component C4, Ca = calcium, C-ANCA = cytoplasmic antineutrophil antibody, CH50 = Complement Hemolytic 50% Reference, CK = creatine kinase, Cl = chloride, CMV IgG = cytomegalo virus immunoglobulin G, CMVIgM = cytomegalo virus immunoglobulin M, Cr = creatinine, CRP = C reactive protein, IgG = immunoglobulin G, DD = D-dimer, EBNA = Epstein-Barr Virus Nuclear Antigen, EBVIgG = Epstein-Barr virus immunoglobulin G, Epstein-Barr virus IgM = Epstein-Barr virus Immunoglobulin M, eGFR = estimated glomerular filtration rate, FDP = fibrin/fibrinogen degradation products, Fib = fibrin, HSV IgG = herpes simplex virus immunoglobulin G, HSV IgM = herpes simplex virus immunoglobulin MM, IgA = immunoglobulin A, IgM = immunoglobulin M, IL-6 = interleukin-6, K = potassium, LDH = lactate dehydrogenase, Na = sodium, P-ANCA = perinuclear antineutrophil antibody, PLT = platelet, PT-INR = prothrombin time-international normalized ratio, RBC = red blood cell, Soluble IL2R = soluble interleukin-2 receptor, T-Bil = total bilirubin, T-Cho = total cholesterol, TG = triglyceride, TP = total protein, UA = urine acid, WBC = white blood cell, γ-GTP = glutamyl transpeptidase.

**Figure 2.**
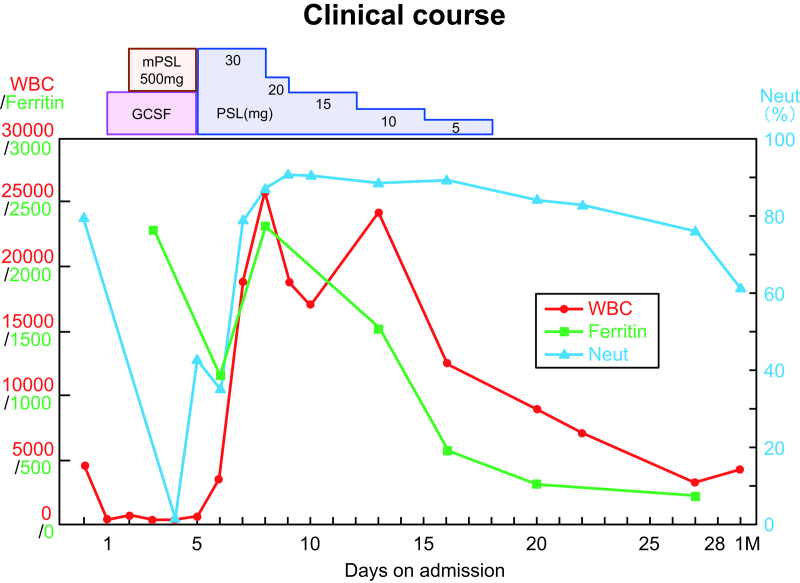
Clinical course. Dynamic changes of the white blood cell counts, neutrophils and ferritin. Fer = ferritin, GCSF = granulocyte stimulating factor, mPSL = methypredonisolone, neut = neutro cell, WBC = white blood cell.

The patient presented with high fever and elevated inflammatory markers, with subsequent evidence of HLH characterized by elevated ferritin, cytopenia, and high levels of soluble IL-2 receptor (sIL2) (Table 1). Bone marrow aspiration showed hemophagocytosis (Fig. 1) but no malignancy. We suspected that HLH was associated with Comirnaty^®^based on the HLH-2004 diagnostic criteria (fulfilling four out of the eight criteria).^[8]^ On the second day of admission, pulsed intravenous (IV) methylprednisolone (500 mg/day for 3 consecutive days) and granulocyte-colony stimulating factor was started, followed by oral prednisolone (30 mg once daily). The patient’s temperature normalized within 12 hours of steroid initiation, and concurrent symptomatic and biochemical improvements were observed (Fig. 2).

## 3. Discussion

An 85-years-old Japanese female with with a 10-years history of nephorosclerosis and hypertension developed fever and nonspecific fatigue 12 days after the first COVID-19 vaccination with BNT 162 b2 COVID 19. To our knowledge, this is a rare case reported in the literature, and only a few studies have reported HLH after receiving the COVID-19 vaccine.^[5–7,9–13]^ It, therefore, it is important to document this condition in order to reiterate the occurrence of such events. Looking at the same scenario from a different angle, it has to be pointed out that rare vaccination events are important, as they help to identify and treat a small number of cases that react in this manner. However, they should not be used to diminish the well-documented safety profile of the mRNA vaccine against COVID-19, which has been widely administered and shows good immunogenicity, tolerance, and high efficacy in inducing immune responses against SARS-CoV-2.^[2,3]^

In COVID-19 patients, secondary HLH and the cytokine storm may be responsible for the unexplained progressive fever, cytopenia, ARDS, neurological and renal impairment.^[4]^ Therefore, mast cell activation syndorome should be considered in a patient with COVID-19 with signs of rapid deterioration of clinical and laboratory-derived parameters.^[4]^ We could not estimate the natural killer cell activity as per the HLH-2004 pediatric diagnostic criteria and the CD163.^[4]^ It seems that we could cover the diagnostic criteria depending on the cell activation value. In this clinical scenario, it is not prudent to wait for haemophagocytosis to initiate treatment. However, a bone marrow biopsy, when available, should be performed as the finding of hemophagocytosis in the bone marrow may help to justify the choice of therapeutic options as sCD25, which serves as a marker of T cell activation, and sCD163, as a marker of hemophagocytosis, is specific for the occurrence of HLH.^[4]^

We estimated the levels of mast cell activation factors, namely ferrtin, IL-2R, and IL-6, and performed bone marrow aspiration. Based on these reports, we initiated PSL therapy during the early phase of the disease. As patients with HLH are frequently critically ill, achieving an accurate and rapid diagnosis is optimal for the best patient outcomes.^[14]^

In the present case, the patient’s age should be considered. The “normal” ratio of CD4:CD8 cells becomes much higher in older age groups, due to a significant decrease in CD8 T cells. Aging also causes a loss of T cell receptor diversity in both CD8 and CD4 cells and reduces T cell survival overall. B cell numbers remain more consistent with age; however, due to reduced expression of select proteins in old age, fewer functional antibodies are produced.^[15]^

The second significant factor in this patient was the presence of chronic kidney disease (CKD) due to nephroscelorosis. The impairment of antigen-presenting ability makes the immune system of CKD patients unable to recognize the pathogen and activate downstream adaptive immunity; therefore, CKD status and advanced age might influence the efficacy of the vaccine.^[16]^

In our patient, the serum erythropoietin level was within the reference range. In the kidney, erythropoietin is produced by interstitial fibroblast-like cells that surround the renal tubules, and insufficient production of erythropoietin can lead to the dysregulation of various fundamental functions in patients with CKD.

Early recognition of HLH and its severe complication of cytokine storm is possible only using a set of diagnostic criteria and by an understanding of the strengths and weaknesses of these criteria in order to utilize them efficiently.

Most importantly, this case report should not be considered as a reason to avoid vaccination, since vaccine campaigns are still the most promising method to combat the COVID-19 pandemic.^[2,17]^ Nevertheless, this report indicates that it is crucial to exclude the presence of other common viruses before COVID-19 vaccination. Patients with underlying conditions should be carefully monitored for suspected symptoms and signs after vaccination. Further studies addressing the performance of these panels of tests and the criteria used to aid in the diagnosis of conditions leading to adverse reactions or incidents in the setting of the COVID-19 vaccination are necessary.

## 4. Conclusion

CKD status and advanced age might influence the efficacy of the vaccine, and patients who fall into this category need to be monitored cautiously to prevent unfavorable outcomes.

## Acknowledgements

The authors thank Mariko Ogi and Tomoko Yoshii for their technical assistance with immunohistochemistry.

## Author contributions

**Conceptualization:** Hiroaki Yokomori.

**Data curation:** Hiroaki Yokomori, Yoshitaka Shimada, Shinya Okada.

**Project administration:** Hiroaki Yokomori, Yoshitaka Shimada, Yasushi Nagaba.

**Software:** Hiroaki Yokomori.

**Supervision:** Shinya Okada, Yasushi Nagaba.

**Writing – original draft:** Hiroaki Yokomori, Yoshitaka Shimada.

**Writing – review and editing:** Hiroaki Yokomori, Yasushi Nagaba, Hiroyuki Ogawa, Kaori Ehara, Shinya Okada.

## References

[1] Wiersinga WJ Rhodes A Cheng AC. Pathophysiology, transmission, diagnosis, and treatment of coronavirus disease 2019 (COVID-19): a review. JAMA. 2020;324:782–93. 32648899 10.1001/jama.2020.12839

[2] Baden LR el Sahly HM Essink B. Efficacy and safety of the mRNA-1273 SARS-CoV-2 vaccine. N Engl J Med. 2021;384:403–16. 33378609 10.1056/NEJMoa2035389 PMC7787219

[3] Polack FP Thomas SJ Kitchin N. Safety and efficacy of the BNT162b2 mRNA Covid-19 vaccine. N Engl J Med. 2020;383:2603–15. 33301246 10.1056/NEJMoa2034577 PMC7745181

[4] La Rosée P Horne A Hines M. Recommendations for the management of hemophagocytic lymphohistiocytosis in adults. Blood. 2019;133:2465–77. 30992265 10.1182/blood.2018894618

[5] Ai S Awford A Roncolato F. Hemophagocytic lymphohistiocytosis following ChAdOx1 nCov-19 vaccination. J Med Virol. 2022;94:14–6. 34406660 10.1002/jmv.27279 PMC8426904

[6] Cory P Lawrence H Abdulrahim H. Lessons of the month 3: haemophagocytic lymphohistiocytosis following COVID-19 vaccination (ChAdOx1 nCoV-19). Clin Med (Lond). 2021;21:e677–9. 34862234 10.7861/clinmed.2021-0564 PMC8806304

[7] Tang LV Hu Y. Hemophagocytic lymphohistiocytosis after COVID-19 vaccination. J Hematol Oncol. 2021;14:87. 34088334 10.1186/s13045-021-01100-7 PMC8177256

[8] Soy M Atagündüz P Atagündüz I. Hemophagocytic lymphohistiocytosis: a review inspired by the COVID-19 pandemic. Rheumatol Int. 2021;41:7–18. 32588191 10.1007/s00296-020-04636-y PMC7315691

[9] Sassi M Khefacha L Merzigui R. Haemophagocytosis and atypical vacuolated *lymphocytes* in bone marrow and blood films after SARS-CoV-2 vaccination. Br J Haematol. 2021;195:649. 34312842 10.1111/bjh.17660 PMC8444800

[10] Rocco JM Mallarino-Haeger C Randolph AH. Hyperinflammatory syndromes after SARS-CoV-2 mRNA vaccination in individuals with underlying immune dysregulation. Clin Infect Dis. 2021;75:e912–5. 10.1093/cid/ciab1024 PMC8689836 34893818

[11] Caocci G Fanni D Porru M. Kikuchi–Fujimoto disease associated with hemophagocytic lymphohistiocytosis following the BNT162b2 mRNA COVID-19 vaccination. Haematologica. 2021;107:1222–5. 10.3324/haematol.2021.280239 PMC9052929 34965702

[12] Baek DW Hwang S Kim J. Patients presenting high fever with lymphadenopathy after COVID-19 vaccination were diagnosed with hemophagocytic lymphohistiocytosis. Infect Dis (Lond). 2022;54:303–7. 34854350 10.1080/23744235.2021.2010801

[13] Hieber ML Sprute R Eichenauer DA. Hemophagocytic lymphohistiocytosis after SARS-CoV-2 vaccination. Infection. 2022;26:1–6. 10.1007/s15010-022-01786-y PMC8881936 35218512

[14] Kikuchi A Singh K Gars E. Pathology updates and diagnostic approaches to haemophagocytic lymphohistiocytosis. Histopathology. 2022;80:616–26. 34716920 10.1111/his.14591

[15] Soiza RL Scicluna C Thomson EC. Efficacy and safety of COVID-19 vaccines in older people age. Ageing. 2021;50:279–83. 10.1093/ageing/afaa274 PMC7799251 33320183

[16] Hou Y-C Lu K-C Kuo K-L. The efficacy of COVID-19 vaccines in chronic kidney disease and kidney transplantation patients: a narrative review. Vaccines (Basel). 2021;9:885. 34452010 10.3390/vaccines9080885 PMC8402591

[17] Alkandari D Herbert JA Alkhalaf MA. SARS-CoV-2 vaccines: fast track versus efficacy. Lancet Microbe. 2021;2:e89–90. 33659918 10.1016/S2666-5247(21)00034-3 PMC7906641

